# ARF family identification in *Tamarix chinensis* reveals the salt responsive expression of *TcARF6* targeted by miR167

**DOI:** 10.7717/peerj.8829

**Published:** 2020-03-18

**Authors:** Youju Ye, Jianwen Wang, Wei Wang, Li-an Xu

**Affiliations:** 1 Key Laboratory of Forestry Genetics & Biotechnology of Ministry of Education, Co-Innovation Center for Sustainable Forestry in Southern China, Nanjing Forestry University, Nanjing, Jiangsu, China; 2 College of Horticulture and Plant Protection, Yangzhou University, Yangzhou, Jiangsu, China; 3 College of Agriculture, Nanjing Agricultural University, Nanjing, Jiangsu, China

**Keywords:** Auxin response factor, MicroRNA response element, MiR167, Salt stress response

## Abstract

Auxin response factors (*ARF*s) are important transcription factors (TFs) that are differentially expressed in response to various abiotic stresses. The important roles of *ARF*s and small RNA-*ARF* pathways in mediating plant growth and stress responses have emerged in several recent studies. However, no studies on the involvement of *ARF*s in tamarisk trees, which are resistant to salinity, have been conducted. In this study, systematic analysis revealed 12 *TcARF* genes belonging to five different groups in *Tamarix chinensis*. The microRNA response elements of miR160, which belongs to group I and miR167, which belongs to group III, were conserved in terms of their location and sequence. Moreover, digital gene expression profiles suggested that a potential miR167 target gene, *TcARF6*, was rapidly expressed in response to salt stress. Cloning of *TcARF6* revealed that *TcARF6* could be an activation TF with a glutamine-rich region and expression pattern analysis revealed that the expression of *TcARF6* was significantly downregulated specifically in the roots. A significant negative correlation in the expression pattern of tch-miR167/*TcARF6* indicated that this module may play a key role in the response to salt stress. Overall, these results provide basic information on the posttranscriptional regulation of *TcARF6* for future investigations of the *T. chinensis* salt-stress response.

## Introduction

Tamarisk plants exist as shrubs or small trees that are naturally distributed in saline soils of Eurasia, the Mediterranean basin and northern and southwestern Africa (Marlin et al., 2017; Sanz Elorza et al., 2010). *Tamarix chinensis* Lour is one of the most highly salt-tolerant tree species that is well suited for investigating salt-stress-response genes (Liu et al., 2014). The high salt tolerance of tamarisk trees was shown by tolerance limit studies and on 340 mM (2% (m/v)) NaCl treatment for 1 week caused no physiological damage to *T. chinensis* (Wang et al., 2018a). This result indicated that the salt tolerance limit of *T. chinensis* could be greater than the 2.1% carbonate limit reported by Song et al. (2006), the 300 mM NaCl limit of *Tamarix aphylla* and the 250 mM NaCl limit of *Tamarix ramosissima* (Natale et al., 2010). Transcription factors (TFs) coordinate gene expression by activating or inhibiting transcription in response to various abiotic stress signals in plants. In our previous study (Wang et al., 2017b), many TFs of *T. chinensis* were predicted to be differentially expressed under NaCl stress. The functions of some of these TFs, such as basic leucine zipper (bZIP), NAC-domain-containing (NACs), basic helix-loop-helix (bHLH) and WRKY TFs of *Tamarix* spp., were verified to increase salt tolerance when heterologously overexpressed (Zheng et al., 2013; Gao et al., 2013; Ji et al., 2013; Yang et al., 2014; Zhou et al., 2014; Wang et al., 2015, 2017a). Other significantly differentially expressed TFs, such as *ARF*s, which are highly important, may be related to salt tolerance and should be further studied.

*ARF*s play a key role in regulating the expression of auxin response genes by binding to auxin response elements (AuxREs) within their promoters (Tiwari, 2003). *ARF*s in *Arabidopsis* (*AtARF*s) play important roles in vascular tissue development, phototropism of the hypocotyl (Hardtke & Berleth, 2014; Pekker, Alvarez & Eshed, 2005), root cap formation (Su et al., 2016; Wang et al., 2005) and other plant developmental processes via auxin signaling (Li et al., 2016). The stress response of *ARF*s was noticed early and Hannah, Heyer & Hincha (2005) proved that the expression of several *AtARF*s is altered by cold stress. To date, transcriptional responses to various abiotic stresses have been identified in various plant species by genome-wide expression profile analyses of *ARF*s, including those in rice (*Oryza sativa*) (drought, cold, salt stress), banana (*Musa nana*) (cold, salt and osmotic stress), tomato (*Lycopersicon esculentum*) (abscisic acid (ABA), indoleacetic acid (IAA) and gibberellin (GA3) treatments) and *Salvia miltiorrhiza* (methyl jasmonate treatment) (Jain & Khurana, 2009; Audran-Delalande et al., 2012; Hu et al., 2015; Xu et al., 2016a). In addition, it seems that auxin regulates stress-induced responses involved in morphogenesis via small RNA (sRNA)-*ARF* pathways, in which microRNA (miRNA)-*ARF*s and trans-acting small interfering RNA (tasiRNA)-*ARF*s are involved. Several studies have suggested that ARF members of group I, such as *ARF*5, *ARF*6 and *ARF*8, targeted by sRNA or miRNA are associated with abiotic stress responses. For example, by fine tuning the expression of auxin-responsive genes, the tasiRNA-*ARF* pathway has been reported to moderate floral morphogenesis in *Arabidopsis* in response to drought stress (Matsui et al., 2014). In addition, the miR167a-*ARF* (*ARF*6 and -8) pathway mediates plant growth and the response to Pi-starvation stress via homeostasis of reactive oxygen species (ROS) and Pi acquisition in tobacco (Chen et al., 2018). Recently, by increasing ABA and proline contents as well as superoxide dismutase activity, the *ARF*5 gene from sweet potato (*Ipomoea batatas*) was shown to increase tolerance to salt and drought in transgenic *Arabidopsis* (Kang et al., 2018). *Arabidopsis* double mutants of miR160 and miR165/166 indicated that ARFs and HD-ZIP IIIs may play opposite roles in the regulation of leaf development and drought tolerance (Yang et al., 2019). These reports suggest that the expression of *ARF*s is induced or repressed by various abiotic stresses and that sRNA-*ARF* modules play important roles in the crosstalk between auxin and abiotic stress signaling (Sharma et al., 2015).

While the high salt tolerance of *T. chinensis* is noticeable, its molecular mechanism remains elusive. Studies of *ARF* profiles under salt stress in a variety of crop species and miRNA-*ARF* pathway associations with salt tolerance suggested that differentially expressed *ARF*s might also play important roles in the salt tolerance mechanism of *T. chinensis*. There have been no studies on *ARF*s in *Tamarix* spp.; thus, we aimed to screen the salt-stress-responsive ARF genes in *T. chinensis* by transcriptome-wide identification of the members of the *ARF* family, by expression profile construction and by miRNA target prediction. Furthermore, we preliminarily investigated the correlations of miRNAs and *ARF* target expression to determine the posttranscriptional regulatory mechanisms involved in the response to salt stress. Our work could serve as a basis for studying the roles of the miR164-*AtARF* pathway in the salt-stress response and would be helpful for further understanding the salt tolerance mechanism of *T. chinensis*.

## Materials and Methods

### Plant materials

The acquisition of cuttings and salt treatment of one-month-old *T. chinensis* ramets followed the methods in our previous study (Wang et al., 2018a). The roots of the ramets were immersed in 340 mM NaCl solution for 0.5 h, 1 h or 4 h and the roots in the control (CK) group were immersed in sterile water for 4 h. The roots, stems and leaves of the salt-treated ramets and CK ramets were harvested (12 tissues with 3 biological replicates for a total of 36 samples). The plant material needed for each sample was collected from 3 to 5 ramets (36–60 plants in total). All samples were quickly frozen in liquid nitrogen and then stored at −80 °C until RNA and DNA extractions were performed.

### Prediction and identification of ARFs

To explore the role of the *ARF* genes in *T. chinensis*, the members of the *TcARF* gene family were predicted from the RNA sequencing (RNA-seq) data (Wang et al., 2017b). Using the hidden Markov model (HMM) prediction referring to the Pfam profile of the *ARF* family (PF06507, http://Pfam.sanger.ac.uk/), we predicted *TcARF* transcripts from the assembled transcripts of the RNA-seq data (Wang et al., 2017b). The proteins of the predicted *TcARF*s were verified by the Plant Transcription Factor Database (http://planttfdb.cbi.pku.edu.cn/) via BLASTP. We used an E-value cutoff of 1E−2 for the HMMER search and 1E−4 for the BLASTP identification.

### Phylogenetic analysis

The conserved sequences (the B3 domain and the AUX_resp domain) of the *TcARF*s and *AtARF*s from *Arabidopsis* (Table S3) were used to construct a phylogenetic tree on the basis of the maximum likelihood method. The sequences of the *AtARF*s were downloaded from The *Arabidopsis* Information Resource database (https://www.arabidopsis.org/). Multiple sequence alignments of the conserved domains (B3 and AUX_resp domains) within the *ARF* proteins were carried out via ClustalX. A phylogenetic analysis was subsequently performed via the neighbor-joining method by MEGA 7, with 1,000 bootstrap replicates.

### Digital gene expression profile analysis

A DGE profile of the tch-miR160 and tch-miR164 *TcARF*s in the *T. chinensis* roots was constructed on the basis of the normalized transcripts per kilobase million (TPM). The TPM was calculated according to the RNA-seq data (Wang et al., 2017b) and the small RNA sequencing (sRNA-seq) data (Wang et al., 2018a).

### Sequence cloning

Genomic DNA was extracted from newly expanded leaves via a DNeasy Plant Mini Kit (QIAGEN, Hilden, Germany). Total RNA was subsequently extracted via an RNAprep Pure Plant Kit (Tiangen, Beijing, China) (Liu, Sun & Xu, 2018). Full cDNA sequences of *TcARF6* were identified according to the manual provided with a SMARTer Rapid Amplification of cDNA Ends (RACE) 5′/3′ Kit (Clontech, Mountain View, CA, USA), with slight modifications (Wang et al., 2018a; 2018b; Cheng et al., 2017). The complete coding DNA sequences (CDSs) of *TcARF1-3*, *5* and *9–12* were identified via sequence amplification with cDNA and introns within *TcARF6* were identified by sequence amplification with DNA. Partial sequences of *TcARF12-14* (those lacking a complete CDS) were included in subsequent analyses. All sequences and primers used are listed in Additional Table S1.

### Expression analysis

The total RNA of the *T. chinensis* roots, stems and leaves was reverse transcribed via PrimeScript RT Master Mix (Takara, Dalian, China) to synthesize first-strand cDNAs. Primers (Tm of 59–60 °C) for *TcARF*6 and the reference gene were designed by Oligo 7 software to amplify the gene-specific PCR products whose length ranged from 70 to 150 bp. Three technical replicates were included; each replicate consisted of a 20 μl mixture that consisted of 10 μl of SYBR Premix Ex Taq (Takara, Dalian, China), three μl of 50× ROX Reference Dye II, 0.2 μl (10 μM) of each primer, two μl of cDNA and 4.6 μl of H_2_O. Quantitative real-time PCR (qRT-PCR) was performed by a Viia 7 Real-Time PCR System according to the manufacturer’s protocol (Wang et al., 2018b). The thermocycling conditions were the same as those reported by Liu, Sun & Xu (2018) and Liu et al. (2018). For quantification of miRNA, 500 ng of the total RNA from the roots was reverse transcribed via PrimeScript RT Master Mix in conjunction with a manually designed stem-loop primer. The qRT-PCR and analysis methods were the same as those mentioned above. The transcription initiation factor (TIF) gene was used as a reference gene. All primers used are listed in Additional Table S2 and the relative expression level was calculated with the 2^−∆∆CT^ method.

## Results

### Identification of ARF family members in *T. chinensis*

Twelve members were identified as belonging to the *TcARF* family. The *TcARF* proteins, which ranged from 624 (*TcARF*9) to 966 (*TcARF*5) amino acids (AAs) in length, had a predicted molecular mass of 73.3 (*TcARF11*) to 105.9 (*TcARF5*) kDa. All 12 *TcARF*s contained conserved B3 and AUX_resp domains. Four *TcARF*s containing microRNA response elements (MREs) were determined to be potential targets of tch-miR167 or tch-miR160 (Table 1).

**Table 1:** Identification of *ARFs* in *Tamarix chinensis*.

Gene	Source	CDS (bp)	MRE	MRE location (bp)
*TcARF1*	PCR	2,031		
*TcARF2*	PCR	2,553		
*TcARF3*	PCR	2,130		
*TcARF4*	Prediction	1,698*		
*TcARF5*	PCR	2,901		
*TcARF6*	RACE	2,631	miR167	2,367–2,387
*TcARF7*	Prediction	1,584*		
*TcARF8*	Prediction	2,340*	miR167	CDS
*TcARF9*	PCR	1,872		
*TcARF10*	PCR	2,091	miR160	1,344–1,364
*TcARF11*	PCR	2,007		
*TcARF12*	PCR	2,094	miR160	1,320–1,340

**Note:**

* Means partial CDS.

**Figure 1:**
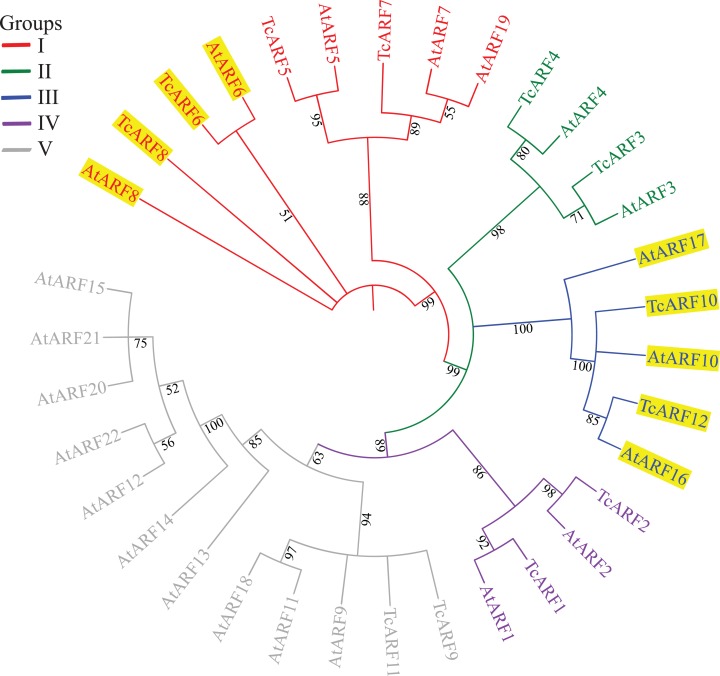
Phylogenetic analysis of *Tamarix chinensis* and *Arabidopsis ARF* proteins. The numbers on each branch indicate bootstrap values and the yellow background indicates miRNA-targeted *ARFs*.

To identify the subfamilies, a phylogenetic analysis of the *TcARF* family members was conducted (Fig. 1). The topological structure and corresponding *ARF* members obtained were identical to those of maize (*Zea mays*), sorghum (*Sorghum bicolor*) and rice (Wang et al., 2010a, 2007; Xing et al., 2011). All of the *ARF* genes clustered into five groups (Fig. 1). Groups I, II, III, IV and V correspond to the *ARF*5/6-like group (*AtARF*5-8, 19), *ARF*3/4 group, *ARF*16/17 group (*AtARF*10, 16 and 17), *ARF*1/2 group and *ARF*9/11-like group of *Arabidopsis*, respectively. The deduced protein length and molecular weight of the *TcARF* proteins in each group varied little (Table S3). The potential *TcARF*s targeted by miRNAs belong to groups I and III, which was in agreement with the miRNA-*ARF* modules in *Arabidopsis*. The *ARF*s targeted by miR167 belonged to group I and encoded relatively large-molecular-weight proteins. All miR160-targeted *ARF*s clustered into group III and the MRE sequences were highly conserved in this group.

### Expression profiles and MREs of ARFs targeted by miRNAs

**Figure 2:**
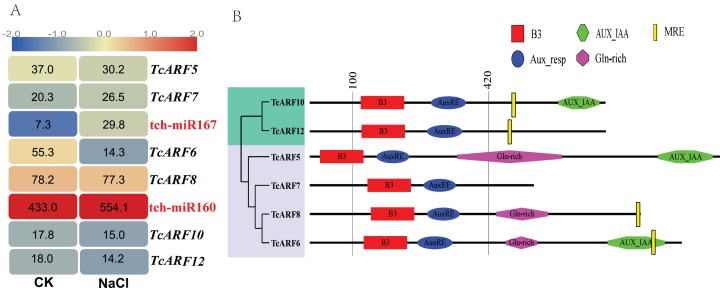
*ARFs* targeted by miRNAs in *Tamarix chinensis*. (A) Digital gene expression profiles of *ARFs* and miR160/167 in *T. chinensis* roots. A heat map was generated on the basis of the normalized Log2 (TPM), which is represented by the red-blue gradation. The columns represent the 0 h and 0.5 h salt treatments (340 mM NaCl). The rows represent *ARF* genes targeted by miRNAs except *TcARF*5 and *TcARF*7. The numbers in the heat map are TPM values. (B) A schematic diagram of MRE locations. The lines show the relative positions of the domains and the numbers indicate the positions of the AA residues. The B3, Aux_resp and CTD domains; the Gln-rich region; and the MRE are represented by different geometric shapes.

DGE profiles were constructed to analyze the expression of *ARF*s in groups I and III (Fig. 2A). In group III (miR160-*ARF* module), the abundance of miR160 resulted in an expression level that was greater for this miRNA (TPM > 200) than for miR167 and the potential targets the former (*TcARF*10/*TcARF*12 of) exhibited a lower abundance (TPM < 20). In group I (the miR167-*ARF* module), the DGE profile suggests that miR167 is upregulated and that the expression of TcARF6 is downregulated (~4-fold). The negative correlation indicated that miR167-*ARF*6 could respond to salt stress, which was further tested with qRT-PCR.

The conserved domains were identified via the NCBI Conserved Domain Search Service (Fig. 2B). For the *ARF*s in groups I and III, the glutamine-rich (Gln-rich) region and the MREs are specific motifs. All members except *TcARF*7 in group I contain a Gln-rich region. The presence of sequences similar to those of the Gln-rich regions of *AtARF*s (e.g., *AtARF6*, *AtARF8*) indicated that these *ARF*s are inhibitory TFs (Wu, Tian & Reed, 2006; Su et al., 2016; Nagpal et al., 2005; Tabata et al., 2010). The miR160 MREs of *TcARF*10/12 were located within the C-terminal region of the Aux_resp domain (~80 AA apart) and encoded the conserved motif GXQGAR. The miR167 MREs were also located within the C-terminal region. The MRE of *TcARF*6 overlapped with the AUX/IAA domain, which was absent in *TcARF*8. The MRE of *TcARF*6 and 8 encoded the conserved motif GSGWQL.

### Cloning of TcARF6 and expression analysis of tch-miR167/TcARF6

**Figure 3:**
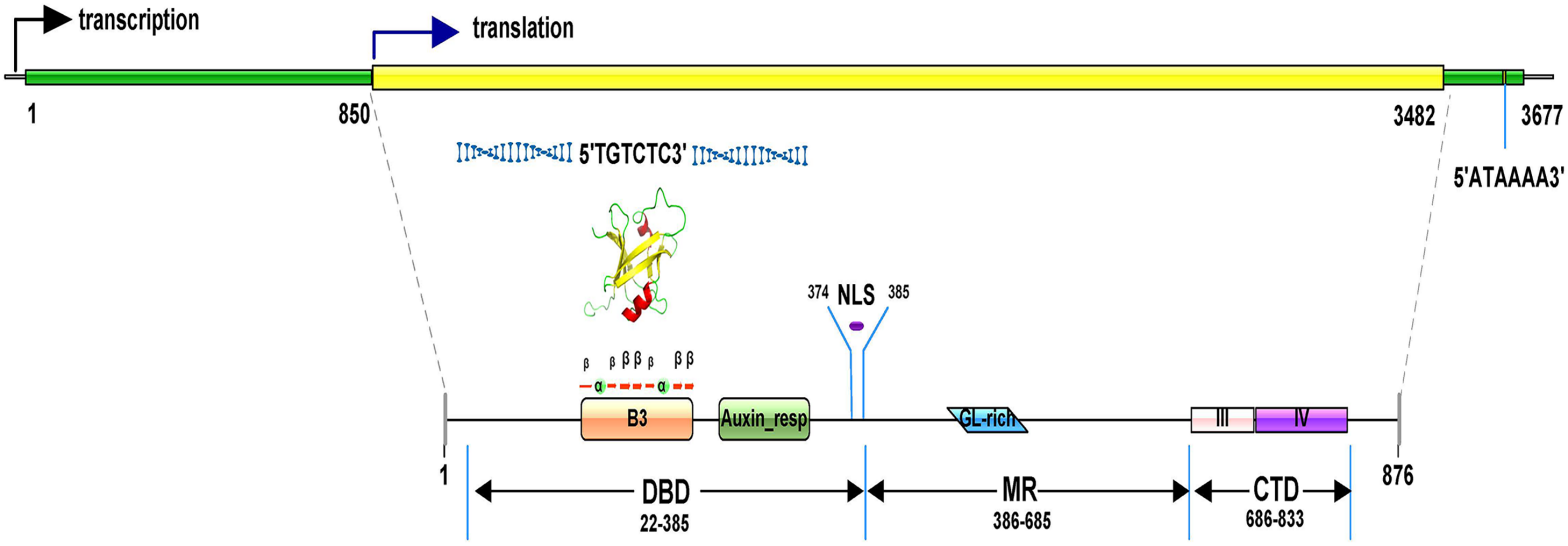
Gene and predicted protein structures of *TcARF6*. In the *TcARF6* gene structure with no introns, the green lines represent the 5′-UTR and 3′UTR. “ATAAAA” is the poly-A signal. The yellow line represents the 2,631 bp open reading frame. In the *TcARF6* protein structure, rectangles of different colors represent different domains. The rhomboids represent the Gln-rich region and the purple line represents the nuclear localization signal. In the B3 domain, α represents an α-helix, and β represents a β-sheet. The tertiary structure represents the spatial conformation of the B3 conserved motif. “TGTCTC” is the promoter recognition site.

Expression profile analysis revealed that *TcARF6* was a candidate responsive gene. Gene cloning and expression analysis of tch-miR167/*TcARF*6 were subsequently performed for validation. The 3,708 bp full cDNA of *TcARF6* was obtained by RACE and included an 854 bp 5′-untranslated region (UTR) and a 226 bp 3′-UTR with a polyadenylation (poly-A) signal (50 bp before the poly-A tail). When the CDS was compared with the genome sequence, it was determined that the *TcARF6* gene structure had no introns (Fig. 3).

The *TcARF*6 protein had typical *ARF* domains (B3, AUX_resp) in the DNA-binding region (DBD) and an AUX/IAA domain in the carboxy-terminal dimerization (CTD) region (Fig. 3). Specifically, the GL-rich motif (462–536 AA, 33% glutamine) in the middle region (MR) was similar to the activation domain (AD) of members in *Arabidopsis* (*AtTcARF5*, *6*, *7*, *8* and *19*). The tertiary protein structure of the B3 domain consisting of seven β-sheets and two α-helices was highly conserved in the B3 protein family. These results suggested that *TcARF6* may recognize AuxREs by binding to the TGTCTC motif. A predicted nuclear localization signal (NLS) was located at the DBD and MR boundary. The CTD consisted of 148 AA residues that encompassed domains III and IV. Domain IV of *TcARF6* is more conserved than domain III; the MRE was located in domain IV (Fig. 3). Only a short deletion from 614 to 684 AA was found from the protein sequence collinearity with *AtARF*6. A similar protein sequence collinearity indicated that *TcARF*6 could be an orthologous gene of *AtARF*6.

**Figure 4:**
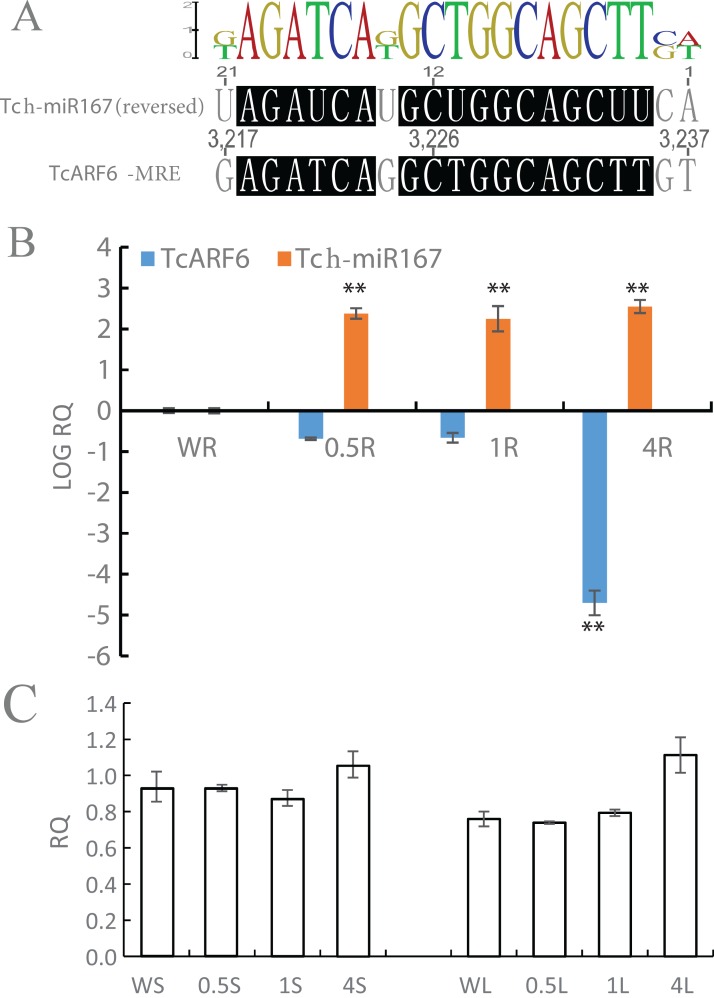
Salt-stress responses of *TcARF6*/tch-miR167 in *Tamarix chinensis*. (A) Sequence complementary of the miRNAs response elements (MRE) and tch-miR167. The numbers indicate the length of tch-miR167 and location of MRE within *TcARF*6. (B) Relative expression of tch-miR167/*TcARF*6 in roots of *T. chinensis*. The *Y*-axis represents the log_2_-transformed relative expression. (C) Relative expression of *TcARF*6 in the aboveground parts of *T. chinensis*; R, S and L represent the roots, stems and leaves, respectively. W (WR, WS, WL) represents tissues under water; 0.5, 1 and 4 represent salt treatments of 0.5 h, 1 h and 4 h, respectively. **Indicates the significance (0.05) by *T*-test compared with the control group.

The 3217–3235 nt of *TcARF6* mRNA (the MRE) was perfectly complementary to the 3–20 nt of tch-miR156, with the exception of one mismatch (the 14th nucleotide of tch-miR156) (Fig. 4A). This indicated that the abundance of *TcARF6* RNA would decrease by mRNA cleavage induced by tch-miR167. Analysis of the expression of *TcARF6* and miR167 in different tissues under 0.5 h, 1 h and 4 h salt stress treatments (Fig. 4B) revealed that *TcARF6* exhibited tissue-specific expression and that its expression was correlated with that of miR167. In the stems and leaves, the expression of *TcARF6* remained relatively stable during the 0–4 h salt treatment (a slight change occurred between 0.65 and 1.11). In the roots, the expression of *TcARF6* was slightly downregulated during the 0.5–1 h salt treatment and sharply downregulated (~25 times) until 4 h (Fig. 4C). Interestingly, the fold change of the upregulated miR167 expression corresponded to that of the *TcARF6* expression. The degree of negative correlation of expression between miR167 and *TcARF6* seemed to increase with prolonged salt stress.

## Discussion

### *ARF* family members in *T. chinensis*

The number of *ARF*s varies among plant species and can be roughly divided into two categories: plant species with more than 20 *ARF*s, such as poplar (35), tobacco (42) and soybean (56) and those with less than 15 *ARF*s, such as *Carica papaya* (11), peanut (10) and cannabis (13) (Li et al., 2016; Kalluri et al., 2007; Wang et al., 2007; Liu et al., 2015; Xu et al., 2016b; Zhang et al., 2017). In this study, 12 *TcARF*s were identified that belong to the category with fewer members. *TcARF*s are grouped into groups I–V, as are most plant *ARF* families. Eighty-three percent of *TcARF*s and *AtARF*s (10/12) are homologous. The high degree linear homology between the *TcARF*s and *AtTcARF*s suggested that the *ARF* family members were linearly correlated in the whole genome after separation from *Arabidopsis* during *Tamarix* evolution. In species with more than 20 *TcARF*s, such as *Populus* and *Salix*, the whole genome had duplicated, thus expanding the number to twice that in *Arabidopsis* (Kalluri et al., 2007).

Introns are the noncoding portions of genes and play a large role in alternative splicing of pre-mRNAs. *ARF*s compose a family of intron-rich genes (Finet et al., 2010; Kalluri et al., 2007). Apart from *Arabidopsis* members in group III (the *ARF*16/17-like group) having relatively few introns (1–3), members in the other four groups have a relatively large number of introns (even more than nine introns). Introns from rice and poplar *ARF*s have been described and the number and structure of introns in the *TcARF*s of the same group are similar (Kalluri et al., 2007; Wang et al., 2007). Forty-nine percent of *Arabidopsis* intron-containing genes are regulated by alternative splicing at the posttranscriptional level in the salt stress response, but most differentially alternatively spliced genes (DAS) are not differentially regulated at the transcriptional level (Ding et al., 2014). Our cloning of the genomic sequences of other *TcARF*s (several ARFs are still being tested, unpublished data) indicated that most *TcARF*s should be intron rich and we suspected that these ARFs could be DASs with stable expression at the transcription level.

*TcARF6* belongs to group I, whose members are associated with an abundance of introns. The intronless structure of the *TcARF6* gene is rare in the *TcARF* family. Only the smallest *SmARF12* (345 AAs, 37.78 kDa) of the 25 *ARF*s from *S. miltiorrhiza* has been reported to be intronless. *SmARF12* belongs to group III and is predicted to localize to the chloroplast rather than the nucleus (Xu et al., 2016a). This may be due to the loss of introns, including NLSs and flanking introns, within the members of group III. However, *TcARF6*, which has a long CDS region and a typical NLS, is considered a macromolecule. Though the long gene length of *TcARF6* differed from that of most intronless genes encoding small proteins, its characteristic rapid expression in response to salt stress corresponded to the response to stress of the intronless genes (Zhang et al., 2014; Yan et al., 2014; Jeffares, Penkett & Bahler, 2008). Genes that respond rapidly to stress have significantly low intron densities throughout the plant genome, suggesting that introns can delay regulatory responses (Jeffares, Penkett & Bahler, 2008; Zhang et al., 2014; Yan et al., 2014). We speculated that the intronless *TcARF6* gene was selected from an intron-containing ancestral gene or paralogs against the requirement for rapid adjustment for survival against environmental challenges during evolution.

### Regulation of TcARF6 under salt stress

Many global expression profiles of plants under abiotic stress suggest that the salt- or drought-induced regulatory pattern of *ARF*s is complex (Die, Gil & Millan, 2018). Different expression patterns can be presented by different members in the same tissue or by the same members in different tissues. Under salt and drought stress, the expression of most of the 23 rice *ARF*s was downregulated, while that of several *ARF*s was upregulated (Li et al., 2016; Luo, Zhou & Zhang, 2018). Under severe salt stress, the expression of all 25 *ARF*s of sorghum was significantly downregulated in the roots, while that of many members was upregulated in the leaves (Wang et al., 2010b). The number of *ARF*s expressed in response to stress greatly differs among species. The expression of only 4 of 28 *ARF*s in chickpea (*Cicer arietinum*) was significantly induced in the roots. The *TcARF* expression profile in which a low percentage of *ARF*s is regulated, such as the situation in chickpea, suggested that only *TcARF6* responds to salt stress. qRT-PCR further revealed that the expression of *TcARF6* was significantly downregulated in the roots but remained stable in the aboveground parts. Though the expression was inhibited by the action of several homologous genes of *TcARF6*, such as salt-inhibited *AtARF6-8*, *SlARF6/7* of tomato and *MnARF6* of banana (Kumar, Tyagi & Sharma, 2011; Audran-Delalande et al., 2012; Hu et al., 2015; Su et al., 2016), root tissue-specific expression patterns were rare. These results indicate that *TcARF*6 may play a unique role in the root response to salt stress.

Recent studies suggest that miR167-*ARF*s can link stress signals to auxin signal transduction (Xi et al., 2018). According to the expression profile analysis, though the abundance of tch-miR167 was not high, its upregulation was negatively correlated with miR167-*ARF*6. Further, qRT-PCR proved that the fold change of upregulated expression increased with salt stress duration. The increasing correlation between the temporal expression profiles of miR167-*ARF*6 was in agreement with canonical miRNA-*ARF* interactions, such as those involving the abiotic stress regulatory module MIR167a-*ARF6*(*8*) in *Arabidopsis* and tobacco (Chen et al., 2018). The salt tolerance role of *ARF*5 (homologous to *ARF*6) in sweet potato has been functionally verified in transgenic *Arabidopsis* and is associated with ABA signaling (Kang et al., 2018). According to the results of our comparative analysis and predicted transcriptional inhibition characteristics, we suspected that *AtARF*6 increases salt tolerance to some extent via posttranscriptional regulation of miR167. In addition, AUX/IAA family members generally present opposite expression patterns under the same abiotic stress conditions. These findings suggest that salt stress may indirectly affect the *ARF*-AUX/IAA interaction, resulting in more complex regulation of *ARF*s. Additional studies on the roles of *TcARF6* under stress should focus on the verification of molecular interactions between miR167 and downstream AUX/IAA elements.

## Conclusions

In the current study, a transcriptome-wide analysis of the *TcARF* gene family was performed. In total, 12 *TcARF* genes were identified via analyses of phylogenetic relationships, conserved domains/motifs and expression profiles in plants under salt stress. Only the expression of *TcARF*6 with a miR167 MRE was significantly downregulated under stress. Further cloning and pattern analysis indicated that *TcARF*6, whose expression is opposite that of tch-miR167, plays a role in the posttranscriptional regulation of the *T. chinensis* salt-stress response. This study provides comprehensive information on the *TcARF* gene family and will aid in investigations of the salt-stress response of *TcARF*s.

## Supplemental Information

10.7717/peerj.8829/supp-1 Supplemental Information 1 All sequences and primers of ARF family in this study. Primers of ARFs cloning; Primers of qRT-PCR; Protein feature of ARFs in phylogenetic analysis.

## Additional Information and Declarations

### Data Availability

The following information was supplied regarding data availability: All primers of this study are available in the Supplemental Files.
